# Diagnostic Challenges in Difficult-to-Localize Insulinomas: A Case Report and Review of Literature

**DOI:** 10.3390/diagnostics14151600

**Published:** 2024-07-25

**Authors:** Nikica M. Grubor, Nikola N. Grubor, Marjan Micev

**Affiliations:** 1 Department for HBP Surgery, Clinic for Digestive Surgery, University Clinical Centre of Serbia, Koste Todorovića 6, 11000 Belgrade, Serbia; gruborn13@gmail.com; 2 Department for Surgery with Anesthesiology, Faculty of Medicine, University of Belgrade, Dr Subotića 8, 11000 Belgrade, Serbia; 3 Institute for Medical Statistics and Informatics, Faculty of Medicine, University of Belgrade, Dr Subotića 15, 11000 Belgrade, Serbia; 4 Department for Pathology, Clinic for Digestive Surgery, University Clinical Centre of Serbia, Koste Todorovića 6, 11000 Belgrade, Serbia; micevm@gmail.com

**Keywords:** insulinoma, Exendin-4, PET/CT, enucleation, hypoglycemia

## Abstract

Non-somatostatin receptor expressing hypovascular insulinomas can be challenging to prove through imaging. This case highlights the utility of a structured approach to molecular imaging in patients with confirmed endogenous hyperinsulinemia. A 54-year-old woman was admitted because of a sudden loss of consciousness. Her relative reported that she complained of dizziness, intense sweating, blurry vision, and upper extremity tingling before becoming unresponsive for 20 min, after which the patient had little recollection of the event. She experienced similar episodes of shorter duration, trouble recalling everyday events, and unintentional weight gain of over 10 kg during the previous two years. Abdominal magnetic resonance imaging (MRI) and multidetector computerized tomography (MDCT) were unremarkable. Selective arterial calcium stimulation significantly increased hepatic venous insulin concentrations when the superior mesenteric and gastroduodenal arteries were stimulated. Technetium-99m (99mTc) octreotide single-photon emission computed tomography (SPECT) did not localize the lesion. Gallium-68 DOTA-Exendin-4 PET/CT acquisition was performed. A single intense 2 cm hyperperfused pancreatic lesion was located anteriorly in the head of the pancreas. Earlier targeted PET/CT imaging and recognition of significant neuropsychiatric symptoms attributable to the patient’s hypoglycemic state might have accelerated the resolution of her condition and obviated the need for unnecessary testing.

## 1. Introduction

Insulinomas are typically benign pancreatic neuroendocrine tumors that secrete insulin inappropriately, leading to episodes of hypoglycemia [1,2]. The incidence of insulinoma is approximately four cases per million individuals per year, making it a rare but important cause of hypoglycemia in the general population [3,4]. The diagnosis is often delayed due to the non-specific nature of the symptoms and the overlap with other more common conditions. In a small number of insulinoma patients (<5–10%), all conventional imaging studies, including endoscopic ultrasound, are negative [5,6]. PET/CT scanning with ^68^Ga-labelled somatostatin analogs has a positive predictive value of 25–31% [7,8]. Consequently, other imaging modalities should be considered in patients with atypical insulinoma features (hypovascularity and inefficient somatostatin receptor expression). A promising noninvasive method is receptor scintigraphy with radiolabeled glucagon-like peptide 1 (GLP-1) receptor analogs, which is shown to be highly sensitive for insulinoma tissue [6,9]. More invasive methods, such as selective intra-arterial injection of calcium and intraoperative ultrasound, are available should other modalities prove inconclusive.

We present the case of a 54-year-old woman who experienced recurrent episodes of severe hypoglycemia manifesting with preferentially neuroglycopenic symptoms (dizziness, intense sweating, blurry vision, transient amnesia), ultimately diagnosed with an insulinoma. Non-somatostatin receptor expressing hypovascular insulinomas can be challenging to prove through imaging. This case covers technical difficulties, the approach to localization, and the added utility of advanced imaging techniques in localizing these neoplasms.

## 2. Case Description

### 2.1. Initial Presentation

A 54-year-old woman was admitted because of a sudden loss of consciousness. Her relative reported that she complained of dizziness, intense sweating, blurry vision, and upper extremity tingling before becoming unresponsive for 20 min, after which the patient had little recollection of the event. She also experienced similar episodes of shorter duration, trouble recalling everyday events, and unintentional weight gain of over 10 kg during the previous two years. The patient had not reported chest pain or shortness of breath before she lost consciousness, and there was no urinary incontinence. The patient’s past medical history was unremarkable. She never used tobacco, alcohol, or other drugs. Her family history was notable only for hypertension in a single first-degree relative. On examination, her temperature was 37.1 °C, pulse 93 beats per minute, blood pressure 153/92 mm Hg, respiratory rate of 20 breaths per minute, and oxygen saturation was 94% while breathing ambient air. She had normal results on cardiac, pulmonary, and abdominal examinations. A comprehensive neurological examination was unremarkable and did not yield any diagnostic leads. The patient did not exhibit abnormalities on cranial computed tomography (CT) imaging or electroencephalography (EEG).

### 2.2. Further Investigations

During her hospital stay, she experienced another sudden episode of upper extremity paresthesia, diaphoresis, and weakness 3 h after a meal. Her blood glucose measured 40 mg/dL (2.2 mmol/L). After intravenous 10% glucose administration, her condition rapidly improved. A 72-h fasting test was performed at a specialized healthcare facility. The results showed that the patient had a morning blood glucose level of 54.1 mg/dL (3.0 mmol/L), insulin levels of 9.7 µIU/mL (67.4 pmol/L), and C-peptide levels of 2.6 ng/mL (0.9 nmol/L). The fasting evaluation was stopped at noon after blood glucose levels reached 43.2 mg/dL (2.4 mmol/L), insulin levels 11.4 µIU/mL (100.0 pmol/L), and C-peptide 2.5 ng/mL (0.8 nmol/L). Ten minutes after administration of 1 mg of glucagon, blood glucose levels rose to 61.3 mg/dL (3.4 mmol/L) and 111.7 mg/dL (6.2 mmol/L) after 30 min. The patient was tested for oral hypoglycemic agents and insulin antibodies, and the results were negative. A clinical diagnosis was made.

The patient was found to have elevated plasma insulin with concomitant low plasma glucose concentration. Negative results for circulating oral hypoglycemic agents and insulin antibodies effectively ruled out oral hypoglycemic agent-induced hypoglycemia and insulin autoimmune hypoglycemia. Sometimes, a hypoglycemic event leads to a diagnosis of malignancy when a significant nonislet cell tumor burden causes low blood glucose. However, this patient appeared well, and elevated plasma insulin and C-peptide concentrations signaled an islet cell pathology. This excess of insulin is consistent with endogenous hyperinsulinemia. To differentiate between genuine insulinoma and β-cell hypertrophy in noninsulinoma pancreatogenous hypoglycemia syndrome (NIPHS), it is crucial to make an effort to identify the precise location of the lesion.

### 2.3. Imaging and Localization

**Figure 1:**
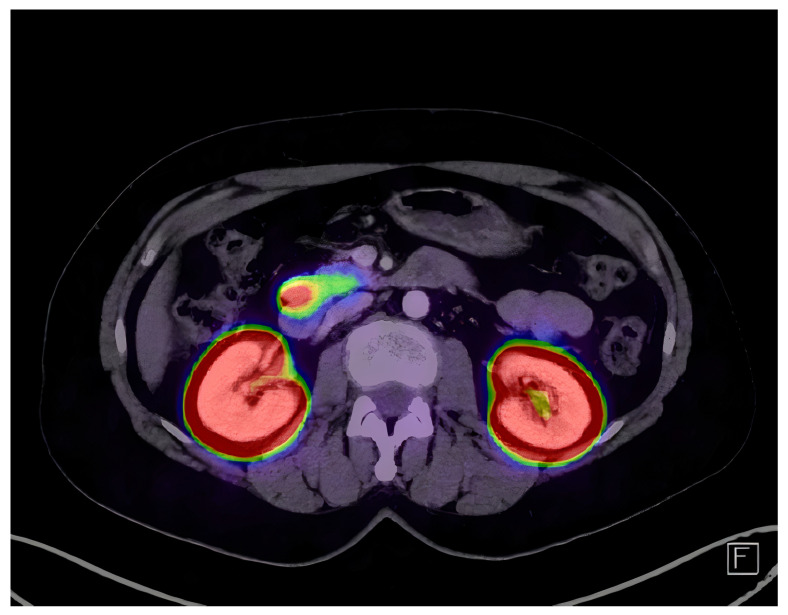
An axial PET/CT image shows a single intense 2 cm hyperperfused pancreatic lesion located anteriorly in the head of the pancreas. There is physiological tracer uptake in the kidneys. Exenedin-4 is a glucagon-like peptide 1 (GLP-1) receptor agonist that can image insulinomas that do not express somatostatin receptors in significant amounts for standard imaging.

Chest radiographs and transabdominal ultrasound were unremarkable. Abdominal magnetic resonance imaging (MRI) and multidetector computerized tomography (MDCT) showed no notable enhancement, with seemingly normal pancreatic parenchyma. No lymph node enlargement was seen. Therefore, the patients received an endoscopic ultrasound (EUS) examination to localize the suspected lesion. Two hypoechoic zones under 5 mm in the pancreatic parenchyma were described but were classified as non-significant. The patient went on to have selective arterial calcium stimulation, which showed significant increases in hepatic venous insulin concentrations when the superior mesenteric artery 392.2 µIU/mL (2723.8 pmol/L), gastroduodenal artery 445.7 µIU/mL (3095.4 pmol/L), and splenic artery 154.1 µIU/mL (1070.22 pmol/L) were stimulated. In addition, technetium-99m (^99m^Tc) octreotide single-photon emission computed tomography (SPECT) did not localize the lesion. Gallium-68 DOTA-Exendin-4 PET/CT acquisition was performed 2 h post slow intravenous injection of 88 MBq ^68^Ga DOTA-Exendin-4. A single intense 2 cm hyperperfused pancreatic lesion was located anteriorly in the head of the pancreas (Figure 1). Pancreatic and biliary ducts were free of contact with the tumor. However, the lesion showed broad contact with the second part of the duodenum.

### 2.4. Treatment and Outcome

**Figure 2:**
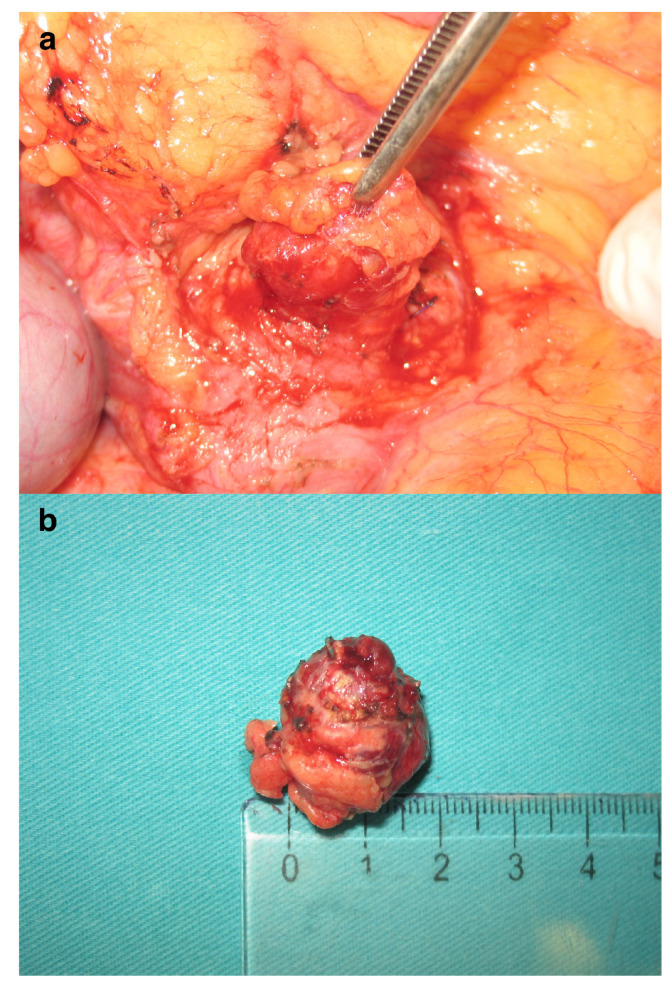
An intraoperative photograph shows the partially dissected tumor located in the pancreatic head (**a**). Once the tumor was freed from all nearby vessels and confirmed not to involve the pancreatic duct, it was excised by blunt tissue dissection with clip closure of all vascular or duct-like structures. This postoperative photograph (**b**) displays a tumor with a clear boundary, surrounded mainly by fibrous and adipose tissue, with some remaining pancreatic tissue interspersed. The approximate size of the enucleated insulinoma was 23 × 22 × 15 mm.

**Figure 3:**
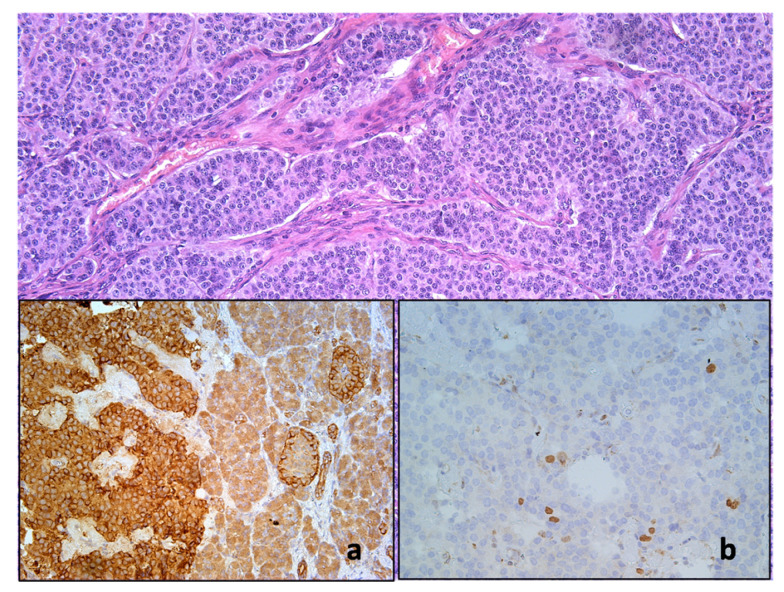
A histological specimen reveals a neuroendocrine neoplasm with a carcinoid structure and a dominant organization of cells in trabecular and ribbon-like patterns. The neoplastic cells display proinsulin immunoreactivity (**a**), similar to the endocrine insulocytes in nearby pancreatic tissue. The measured Ki-67 protein index (**b**) indicates a proliferative activity of 4.7%.

Surgical enucleation of the pancreatic head was performed by careful, mainly blunt tissue dissection with clip closure of any vascular or duct-like structures (Figure 2). No evidence of invasion was found at the site of contact with the duodenum. Histopathological examination of the excised specimen showed a well-differentiated neuroendocrine tumor consistent with a proinsulin-secreting insulinoma (Figure 3). The patient recovered without complications. Six years into follow-up, she remains healthy and symptom-free.

## 3. Discussion

### 3.1. Clinical Considerations

Obtaining a detailed medical history is the first step in elucidating the cause of loss of consciousness (LOC) [10]. Syncope is a clinical syndrome in which a period of inadequate cerebral blood flow or oxygen supply causes LOC [11]. The potential causes of LOC in this patient should include both transient and non-transient causes of LOC due to the unusual length of her symptoms. During transient episodes, inadequate cerebral nutrient flow is brief, and normal brain functioning rapidly resumes with no lasting consequences. Transient causes include syncope, seizure, stroke, and psychogenic pseudosyncope, while non-transient causes are related to toxic-metabolic causes and status epilepticus (convulsive and nonconvulsive) or stroke [10]. Loss of consciousness is not a characteristic of most transient ischemic attacks (TIA) or strokes [12]. Even though her symptoms did not include headache, delayed diagnosis of subarachnoid hemorrhage occurs because a significant portion of these patients present as neurologically intact. The symmetric appearance of sensory abnormalities and unintentional weight gain make TIA or stroke less likely and are concerning for a systemic cause. Performing a neurological examination on the patient is crucial in ruling out any possible underlying causes. In patients with confirmed hypoglycemia, it is beneficial to consider whether they appear ill (or are under medication) or are seemingly healthy. Causes of hypoglycemia in the sick or medicated patient include drugs, critical illnesses, hormone deficiency, and nonislet cell tumors; in the healthy-appearing individual, causes include endogenous hyperinsulinism or accidental, surreptitious, or malicious hypoglycemia. Given that this patient appears well and has been under observation, endogenous hyperinsulinism seems likely.

Insulinomas can be single or multiple and benign or malignant. Most cases are sporadic, benign, and solitary (87%), with multiple tumors (7%) and malignant variants (6%) being rarer [3]. Multiple long-running series indicate that the median age of surgical diagnosis is around 50 years, with similar occurrence rates in both sexes [3,4]. The median duration of symptoms before diagnosis is less than 1.5 years, though some patients can be symptomatic for decades [3]. Although various motor, sensory, and mental symptoms have been described with insulinomas, psychiatric and neurologic problems are the most frequent complaints before tumor identification [13]. The neuroglycopenic symptoms of insulinoma include confusion, visual change, and unusual behavior [2,13]. In selected reports, as many as 20% of patients with insulinoma had been misdiagnosed with a neurologic or psychiatric disorder before insulinoma was recognized. Seizure disorder is another common misdiagnosis [2,14]. Neuroglycopenic symptoms directly result from glucose deprivation of the central nervous system, resulting in impaired cognitive function. These chronic hypoglycemic states can lead to neurologic deterioration over time [2,13]. As around 40% of patients may be unaware of any unusual pattern of behavior during a hypoglycemic event, a detailed history needs to be obtained from family members or friends [1,13]. The most common sympathoadrenal symptoms are diaphoresis, weakness, palpitations, and tremors [2,13]. Most patients become symptomatic exclusively in the fasting state—however, a significant portion exhibit both fasting and postprandial symptoms [4]. Patients with severe episodes of hypoglycemia self-manage the condition with frequent meals during the day, often resulting in unintentional weight gain.

### 3.2. Clinical Diagnosis

Hypoglycemia is a condition characterized by abnormally low blood glucose levels, which can lead to a variety of clinical manifestations, ranging from mild neuroglycopenic symptoms such as confusion and dizziness to severe outcomes like loss of consciousness and seizures [15]. Although hypoglycemia is a common complication in diabetic patients undergoing treatment, it is relatively rare in non-diabetic individuals, posing a significant diagnostic challenge. A 72-h fasting test will help distinguish hyperinsulinemia (endogenous or exogenous) from other causes of hypoglycemia. Prolonged fasting will provoke hypoglycemia only if there is a defect in maintaining normal blood glucose levels. Elevations in plasma C-peptide occur when endogenous insulin production is present. Therefore, its measurement distinguishes endogenous from exogenous hyperinsulinemia. The presence of insulin or insulin receptor antibodies can distinguish insulin autoimmune hypoglycemia from insulinoma. Patients with noninsulinoma pancreatogenous hypoglycemia syndrome (NIPHS) have increased plasma insulin, C-peptide, and proinsulin levels [16,17]. NIPHS is endogenous hyperinsulinemia due to islet hypertrophy and nesidioblastosis (new development of islets of Langerhans from pancreatic duct epithelium). A characteristic feature of this disorder is that hypoglycemia occurs two to four hours after a meal [17]. Fasting hypoglycemia is now a recognized characteristic of insulinoma but is rare in NIPHS [4,17,18]. The differential diagnosis in patients with endogenous insulin-mediated hypoglycemia includes insulinoma, NIPHS, oral hypoglycemic agent-induced hypoglycemia, and insulin autoimmune hypoglycemia. The diagnosis of insulinoma is established by demonstrating inappropriately high serum insulin, C-peptide, and proinsulin concentrations without evidence of sulfonylurea use during a spontaneous or provoked episode of hypoglycemia [15]. Her autonomic (sweating, hunger, and paresthesia) and neuroglycopenic (dizziness, weakness, amnesia) symptoms and documented low blood glucose level with successful complete reversal after glucose administration constitute Whipple’s triad, which is a classic presentation of insulinoma [4].

### 3.3. Lesion Localization

Localizing studies should only be performed once endogenous insulin-mediated hypoglycemia has been demonstrated. Due to this patient’s complex presentation, we hoped these imaging studies would prove helpful in determining whether we were dealing with a focal or diffuse process. The spectrum of endogenous hyperinsulinism includes diffuse β-cell hyperplasia/nesidioblastosis, which accounts for a significant percentage of hypoglycemia patients seen at this hospital and makes preoperative localization important [16,19]. Describing pancreatic duct involvement is very important if a focal disease process is found. Blind surgical exploration poses significant risks of missed insulinoma and reoperation.

Due to their abundant capillary network, average functioning pancreatic NETs present as small hypervascular lesions. It is rare for pancreatic NETs to involve the central pancreatic duct, but in scarce circumstances, they may cause appreciable upstream dilatation [20,21]. Pancreatic NETs appear hypointense on T1 and hyperintense on T2 magnetic resonance imaging [20]. Diffusion-weighted MR imaging and apparent diffusion coefficient (ADC) mapping have been reported to provide valuable information in localizing non-hypervascular tumors [22]. Endoscopic US should be considered when CT/MR findings are ambiguous, as this technique shows better sensitivity than CT in detecting insulinomas. However, some insulinomas are not detectable by preoperative endoscopic US, especially if the lesion is isoechoic; the sensitivity of detection will depend on the tumor’s location and the patient’s characteristics.

The hypoechoic zones noted on EUS in our patient could be spurious findings or represent multiple neoplastic foci; overall, the imaging study was inconclusive. Dynamic contrast-enhanced ultrasound can increase the sensitivity of classical B-mode scanning by revealing temporal and spatial patterns of tumor contrast uptake and washout when initial scanning is equivocal [23]. However, the patient went on to receive selective arterial calcium stimulation testing (SACST) with hepatic venous sampling to distinguish between a focal abnormality (insulinoma) and a diffuse process (islet-cell hypertrophy/nesidioblastosis) [16,19]. In patients with insulinoma, the SACST response is positive in one artery unless the tumor is fed by two arteries or the patient has multiple insulinomas scattered throughout the pancreas [5,16,24]. A few patients have aberrant arterial anatomy that may explain multiple areas of response, but this finding was not described in this patient. In patients with islet-cell hypertrophy, positive responses are usually, but not always, observed after injection of multiple arteries. The regionality of the tumor was narrowed to the pancreatic head or neck; however, since testing was not unequivocal, we believe that additional imaging is warranted. ^99m^Tc octreotide SPECT yielded no pertinent findings, and the tumor was considered somatostatin receptor 2 negative. Insulinomas express relatively low levels of subtype 2 somatostatin receptors, making them less likely to be detected with somatostatin-receptor-based imaging [9]. However, many insulinomas have high concentrations of glucagon-like peptide-1 (GLP-1) receptors [25]. Therefore, the patient was scheduled to receive targeted imaging.

The discovery that neuroendocrine tumors homogenously overexpress somatostatin receptors (SSTRs) has been critical to oncologic surgery. SSTR PET/CT is currently the gold standard imaging technique for insulinomas, and SSTR scintigraphy should only be performed when other methods are unavailable [26]. However, PET/CT has poor diagnostic accuracy when imaging insulinomas due to their small size and low SSTR expression [27]. Glucagon-like peptide-1 receptors (GLP-1R) are localized on pancreatic β-cells, overexpressed, and homogenously distributed on benign insulinomas. GLP-1 analog Exendin-4 targets GLP-1R and should be used to visualize benign insulinomas that localize poorly using conventional imaging [25]. In addition to its high diagnostic accuracy (Table 1), Ga-68 DOTA-Exendin-4 PET/CT has the advantage of having a short investigation time (1–2 h) and low radiation exposure [26]. The alternative to localizing insulinoma is the highly invasive procedure of selective intra-arterial calcium stimulation and venous sampling, with associated risks.

### 3.4. Surgical Treatment

At our institution, we prefer open surgical removal of insulinoma, which offers a potentially permanent cure, with rates as high as 98–100% [6,37]. Enucleation should be performed whenever possible to reduce the risk of morbidity associated with extensive pancreatic surgery. Thus, the most critical surgical step is proper preoperative imaging to ensure complete tumor removal. Insulinomas are not always easily palpable intraoperatively, and fracturing tumor tissue during dissection can lead to tumor regrowth and reoperation [19]. Even when the approximate location of the lesion is known, it can be difficult to localize with intraoperative ultrasound [9]. Blind surgical exploration should be avoided in as many cases as possible [19]. Patients who fail initial surgery or develop recurrent disease have a higher probability of a multiple endocrine neoplasia type 1 (MEN1) genotype with multiple tumors (25%) or malignant insulinomas (13%) [3]. Recent reviews of laparoscopic approaches favor open enucleation, with pancreatic duct involvement causing high conversion rates to open surgery and a significant incidence of pancreatic fistula formation [6,38,39]. In a recent review by Rossi et al. [40], the possibility of endoscopic ultrasound radiofrequency ablation of small focal pancreatic NETs in the frail elderly population was raised following several small, relatively successful case series. This novel approach shows promise of symptom relief to those unfit for surgery, with confined disease amenable to the EUS approach. However, the clinical utility of this intervention will be known once more data on long-term follow-up, adverse events, and recurrence rates are produced.

Patients with non-metastatic insulinoma have a long-term prognosis comparable to that of the general population. In individuals without MEN1, the recurrence rates at 10 and 20 years are 5% and 7%, respectively [3,41]. The timeframe of recurrent disease ranges from 4 to 18.5 years after the initial operation. Recurrence rates in MEN1 patients are more frequent, with 10- and 20-year recurrence rates of 21% [3].

## 4. Conclusions

Poor diagnostic test accuracy still poses a fundamental challenge in pancreatic oncology. Surgery is the only curative option when medical management fails, or tumors have significant malignant potential. Even after establishing a biochemical diagnosis, failure to correctly identify the location and extent of oncological disease could significantly increase morbidity and mortality. A steady, structured escalation to more accurate diagnostic methods ultimately resolved this patient’s symptoms. This case highlights the utility of a structured approach to molecular imaging in patients with confirmed endogenous hyperinsulinemia. Earlier targeted PET/CT imaging and recognition of significant neuropsychiatric symptoms attributable to the patient’s hypoglycemic state might have accelerated the resolution of her condition and obviated the need for unnecessary testing.

## Figures and Tables

**Table 1:** True positive rates (sensitivities) for insulinoma detection reported in literature.

	Characteristics	Median True Positive Rate (Range)	References
MDCT	Small (<2 cm), well-defined, hypervascular, rarely hypovascular	63% (40–77%)	[25,28,29,30,31,32,33]
MRI	T2WI hyperintense, T1WI hypointense, ADC map	66% (56–85%)	[25,29,30,31,33,34,34]
SRS	Low expression of SSTR (50–60%)	44% (33–68%)	[7,25,30,31,32,33,35]
EUS	EUS guided FNA, superior in the proximal pancreas, rarely isoechoic	85% (82–93%)	[28,30,33,36]
Exendin-4 PET/CT		94% (85–98%)	[25,31,32,33,35]

## Data Availability

No new data were created or analyzed in this study. Data sharing is not applicable to this article.
